# Screening of antidepressant effective active components of *Pueraria* and investigation of the mechanism

**DOI:** 10.3389/fnut.2025.1694376

**Published:** 2025-11-14

**Authors:** Li Li, Jun-jie Gao, Min Yan, Li Guan, Ming-ming Qin, Kai Ye, Tao Li

**Affiliations:** 1 Department of Clinical Laboratory, The First Affiliated Hospital of Anhui Medical University, Hefei, Anhui, China; 2 Department of Clinical Laboratory, The First Affiliated Hospital of Wannan Medical College (Yijishan Hospital of Wannan Medical College), Wuhu, China; 3 Department of Clinical Laboratory, The Second Affiliated Hospital of Wannan Medical College, Wuhu, China; 4 Department of Hepatology, Wuhu Third People's Hospital, Wuhu, China

**Keywords:** *Pueraria*, depression, network pharmacology, machine learning, neuroprotection

## Abstract

**Background:**

Depression is a prevalent mental disorder, with its incidence rising alongside the increasing pressures of modern social life. Although medications remain a cornerstone of treatment, first-line antidepressants are often associated with significant side effects. *Pueraria*, a plant rich in isoflavonoid active ingredients, has demonstrated neuroprotective effects; however, the specific mechanisms behind its antidepressant components have not been fully elucidated.

**Methods:**

This study employed an integrated approach combining network pharmacology, transcriptomics, and machine learning to explore the mechanisms of *Puerari's* antidepressant active ingredients. Multiple transcriptomic datasets were analyzed, and active ingredients, depression-related genes, and key targets were identified through the GEO, HERB, TCMSP, GWAS, and PDB databases. Molecular docking simulations were used to assess the binding affinity between the key active ingredients (daidzein and methyl p-coumarate) and the primary targets of *Pueraria* extracts. *In vivo* validation was conducted using a chronic mild stress (CMS) mouse model to evaluate the antidepressant effects of daidzein and methyl p-coumarate.

**Results:**

We identified eight signature genes related to both *Pueraria* and depression, with MMP9, MGAM, and CDK5R1 being of particular importance. Molecular docking revealed that daidzein and methyl p-coumarate strongly bind to these three key genes, supporting their neuroprotective efficacy. *In vivo* experiments confirmed that both daidzein and methyl p-coumarate reversed depressive-like behaviors in CMS mice, with daidzein demonstrating a particularly significant antidepressant effect.

**Conclusion:**

*Pueraria*, as a traditional medicinal herb with both food and medicinal uses, shows promising antidepressant potential through its active ingredient daidzein. This not only offers a novel approach for the prevention and treatment of depression but also provides new theoretical perspectives and research pathways for understanding antidepressant mechanisms.

## 1 Introduction

Depression is a highly prevalent psychiatric disorder globally, characterized by persistent low mood, anhedonia, cognitive dysfunction, and somatic symptoms (1). In modern society, where psychological and social pressures are intensifying, the incidence of depression continues to rise (2). According to the World Health Organization (WHO), ~3.8% of the global population is currently affected by depression (3). By 2030, depression is projected to become the leading contributor to the global disease burden (4), highlighting the urgent need for effective preventive and therapeutic strategies. Several hypotheses have been proposed to elucidate the underlying pathophysiology of depression, including the neurotrophic hypothesis, neuroinflammatory hypothesis, monoamine neurotransmitter hypothesis, gut–brain axis dysregulation, synaptic plasticity impairment, and hypothalamic–pituitary–adrenal (HPA) axis dysfunction (5–7). Although pharmacotherapy remains a mainstay in clinical management, commonly prescribed antidepressants—particularly selective serotonin reuptake inhibitors (SSRIs)—are associated with delayed therapeutic onset, treatment resistance, gastrointestinal disturbances, sexual dysfunction, and diminishing long-term efficacy (8, 9). These limitations have prompted increasing interest in alternative or adjunctive therapies derived from natural sources, which offer the advantages of reduced toxicity, fewer side effects, and broader accessibility (10).

*Pueraria* (commonly known as kudzu), a perennial leguminous vine, has been used in traditional medicine across China, Japan, and Southeast Asia for over two millennia. Pharmacological studies have revealed that *Pueraria* is rich in bioactive isoflavones, as well as triterpenoid saponins, polysaccharides, and trace elements (11). These constituents confer diverse biological properties, including antioxidant (12), anti-inflammatory, and neuroprotective effects (13). In recent years, *Pueraria* has demonstrated therapeutic potential in the management of cardiovascular (14) and neurodegenerative diseases (15). Notably, its antidepressant effects have gained increasing attention. For instance, *Pueraria* has been shown to ameliorate depressive-like behaviors in murine models by modulating gut microbiota composition (2). Puerarin, one of its primary components, significantly reduces immobility time in the forced swim and tail suspension tests in ovariectomized mice (16). Moreover, *Pueraria* exhibits a synergistic effect across multiple bioactive components and targets, supporting its use as a multi-targeted therapeutic agent (17). Importantly, *Pueraria* is recognized not only as a traditional Chinese herbal medicine but also as a dietary component. Its root can be processed into products such as kudzu powder and kudzu tea, which are commonly consumed in East Asian cuisine (18). This dual-use nature makes *Pueraria* a promising low-cost, low-risk, and sustainable candidate for adjunctive dietary intervention in depression management.

Previous studies have primarily focused on the neuroprotective effects of puerarin (2, 16, 19). However, the complexity and synergistic nature of *Pueraria* components, combined with the unclear material basis and molecular mechanisms of its antidepressant effects, necessitate further investigation. Screening active constituents and identifying target molecules are crucial for elucidating the molecular mechanisms underlying *Pueraria's* antidepressant activity. In this study, we applied an integrative research framework combining network pharmacology, transcriptomic analysis, and machine learning algorithms to systematically investigate the “multi-component–multi-target–multi-pathway” mechanisms of *Pueraria* and its active compounds. Our objective was to elucidate the molecular basis of *Pueraria's* antidepressant potential and validate its efficacy *in vivo*. Given its neuroprotective properties and medicinal-food duality, *Pueraria* offers a unique advantage as a long-term, mild, and accessible strategy for the adjunctive management of depression.

## 2 Materials and methods

### 2.1 Animal experiments

SPF-grade male C57BL/6J mice were obtained from Ailingfei Biotechnology Co., Ltd. The mice were housed in an animal facility at the Animal Research Center of Wannan Medical College, which maintained a specific pathogen-free environment with a temperature of 22 ± 2 °C, humidity of 50 ± 5%, and a 12-h light/dark cycle. Mice had *ad libitum* access to food and water. All experimental procedures were performed by qualified personnel, and the study was approved by the Animal Ethics Committee of Wannan Medical College (Approval number: WNMC-AWE-2023109).

### 2.2 Drugs and vehicle

*Pueraria* was purchased from Jiangzhong Traditional Chinese Medicine Co., Ltd. (Nanchang, China). Following the protocol described previously (20), 10.0 g of fresh *Pueraria* was soaked in 100 ml of 80% ethanol at 4 °C overnight. After equilibrating at room temperature, the *Pueraria* was extracted with 80% ethanol at 80 °C for 2 h. The supernatant was vacuum evaporated to dryness using a rotary evaporator and stored at 4 °C to obtain *Pueraria* extract (PUE). Daidzein (DD) was purchased from Sigma Chemical Co. (St. Louis, MO, USA), and methyl p-coumarate (MpC) was purchased from SynQuest Labs, Inc. (Alachua, FL, USA). For drug preparation, 10.0 g of each substance was dissolved in 1 ml of anhydrous ethanol and mixed with sterile olive oil. The vehicle solution was composed of a 1:9 ratio of anhydrous ethanol to olive oil.

### 2.3 Chronic mild stress (CMS) model

**Figure 1:**
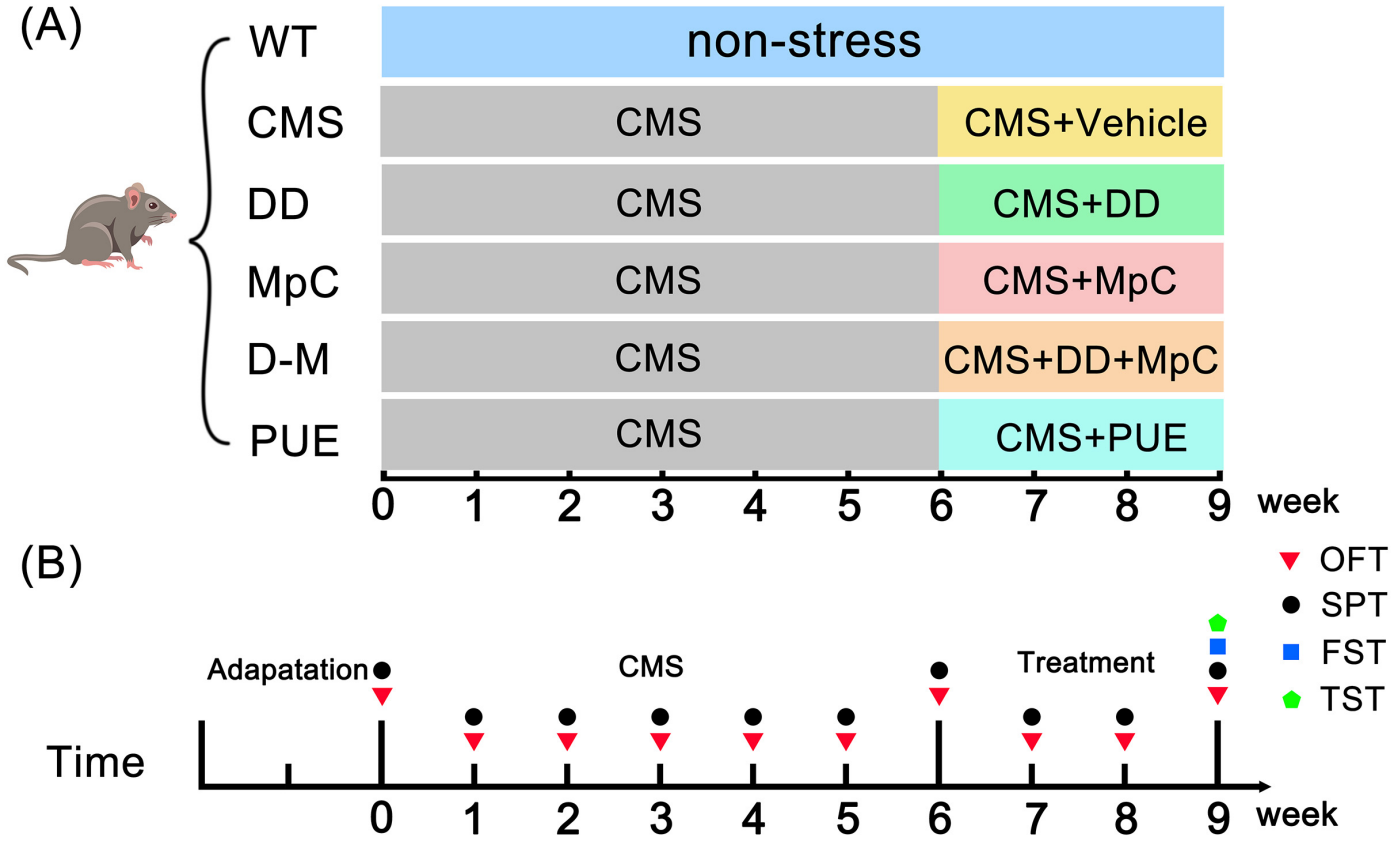
Animal experiment design section. **(A)** Chronic mild stress (CMS) model was established for 9 weeks. CMS mice were divided into five groups: the CMS group, the daidzein (DD) group, the methyl p-coumarate (MpC) group, the daidzein+methyl p-coumarate (D+M) group, and the *Pueraria* extract (PUE) group. In the 6th week, Vehicle, DD, MpC, DD+MpC, and PUE treatments were given. **(B)** Behavioral testing timeline for mice.

Mice were randomly assigned to six groups: Wild-type (WT, *n* = 6), CMS (*n* = 6), DD (*n* = 6), MpC (*n* = 6), daidzein + methyl p-coumarate (D+M, *n* = 6), and PUE (*n* = 6). As described previously (21), mice were acclimatized to the environment for 2 weeks, followed by 6 weeks of exposure to various mild stressors. Stressors included restraint (2 h), wet bedding (24 h), no bedding (24 h), loud noise (12 h), tilted cage (45°, 24 h), strobe lighting (12 h), reversed light/dark cycle (24 h), food restriction (12 h), and tail suspension (2 h). During the final 3 weeks, mice were treated with different drugs (20 mg/kg, qd) while being exposed to CMS, as illustrated in Figure 1A.

### 2.4 Behavioral tests

Behavioral tests were performed to assess depression-like and anxiety-like behaviors in mice using the Open Field Test (OFT), Forced Swimming Test (FST), Tail Suspension Test (TST), and Sucrose Preference Test (SPT) at the time points shown in Figure 1B. A trained observer, blinded to the experimental groups, utilized Supermaze software to record all behavioral parameters.

#### 2.4.1 OFT

The OFT was conducted to assess the mice's spontaneous activity, anxiety-like behavior, exploratory behavior, and motor ability. The mice were placed in an open field box (25 × 25 × 40 cm) divided into nine equal-sized squares. The time spent and distance traveled in the outer and central areas of the arena were recorded over a 10-min period. After each trial, the box was cleaned with 75% ethanol to remove odor cues.

#### 2.4.2 FST

The FST was employed to evaluate depressive states. Mice were individually placed in glass cylinders (50 cm in height, 20 cm in inner diameter) containing 15 cm of water at 23–25 °C for acclimatization. On the following day, mice were again placed in the same environment for 6 min. Behavioral parameters, including immobility time, swimming time, and struggling time during the last 5 min, were recorded via a video camera.

#### 2.4.3 TST

The TST was used to measure depressive-like behavior. Mice were suspended individually by their tails 50 cm above the floor using tape placed 1 cm from the tail's end for 6 min. A video camera recorded immobility and struggle times during the last 5 min.

#### 2.4.4 SPT

The SPT was performed to assess anhedonia in the mice. Mice were individually housed for 3 days with two drinking bottles containing either normal water (A) or 1% sucrose solution (B) for 2 days. The positions of the bottles were alternated every 24 h. Prior to the test, mice were fasted for 24 h. The bottles were reoffered for 2 h, and the total weight of each bottle was measured before and after the test. Sucrose preference was calculated as follows: Sucrose preference=100 % × [B/(A + B)].

### 2.5 Data set acquisition and preprocessing

Six depression-related transcriptome datasets (GSE32280, GSE38206, GSE39653, GSE52790, GSE76826, GSE98793) were obtained from the GEO database (https://www.ncbi.nlm.nih.gov/geo/; accessed on 1 March 2025). The data selection criteria included: human samples (depressed patients vs. healthy controls), original or normalized expression matrices with complete phenotypic information, sample size ≥10, and traceable platform information. The details of the datasets are as follows: GSE32280 (five depressed patients, eight healthy controls), GSE38206 (nine depressed patients, nine healthy controls), GSE39653 (21 depressed patients, 24 healthy controls), GSE52790 (10 depressed patients, 12 healthy controls), GSE76826 (10 depressed patients, 12 healthy controls), and GSE98793 (128 depressed patients, 64 healthy controls). Data were normalized using the limma package (v3.52.4) in R (v4.4.3), and batch effects were corrected using the ComBat algorithm from the sva package (v3.44.0). The data, before and after batch effect correction, were subjected to principal component analysis (PCA) for visualization.

### 2.6 Differential expression and co-expression network analysis

Differentially expressed genes (DEGs) were identified using the limma package with a significance threshold of |logFC| > 0.1 and a corrected *p*-value < 0.05. Gene co-expression networks were constructed using Weighted Gene Co-expression Network Analysis (WGCNA, v1.72). Genes were grouped into modules using the dynamicTreeCut method, with a minimum module size of 60 genes. The correlation between each module's eigengene (ME) and the depression phenotype was calculated. DEGs were integrated with WGCNA module genes via a Venn diagram.

### 2.7 Pharmacologic analysis of *Pueraria* network

The primary active components of *Pueraria* and their targets were extracted from the HERB database (http://herb.ac.cn/; version 2.0, accessed on 5 March 2025) and the Traditional Chinese Medicine Systems Pharmacology Database and Analysis Platform (TCMSP, https://www.tcmsp-e.com; version 2.3, accessed on 5 March 2025). Intersection analysis of *Pueraria* targets with depression-related targets was performed to identify overlapping genes. The protein interaction network was visualized using Cytoscape (v3.9.1).

### 2.8 Functional enrichment analysis

Gene Ontology (GO) functional annotation and KEGG pathway enrichment analysis of the overlapping genes were performed using the clusterProfiler package (v4.4.4). GO analysis covered three main categories: molecular function, cellular components, and biological processes.

### 2.9 Machine learning-based screening of characterized genes

Three complementary machine learning methods are used for feature gene screening. LASSO regression: determining the optimal λ-value and screening the core genes by 10-fold cross validation; SVM-RFE: determining the optimal number of features by 10-fold cross validation to obtain the subset of genes with the highest accuracy; and Random Forest (RF): screening important genes based on the out-of-bag error rate (OOB). Integrate the intersection of the three to obtain the core genes.

### 2.10 SHAP interpretability and Mendelian randomization (MR)

An artificial neural network with six hidden nodes was built based on the machine learning model to assess the association between core genes and depression. Gene importance was quantified using SHAP analysis. Two-sample Mendelian randomization (MR) was employed to test the causal relationship between MMP9 and depression. Exposure data were sourced from the GWAS database, and outcome data were obtained from the Finnish database (including 242,809 healthy controls and 54,733 depression cases). The causal relationship was further confirmed by inverse variance weighting (IVW) analysis.

### 2.11 Molecular docking validation

The three-dimensional structures of MMP9, MGAM, and CDK5R1 were retrieved from the PDB database and preprocessed using PyMOL (v2.5.2). The active components of *Pueraria* (e.g., DD and MpC) were optimized using Avogadro (v1.95.0) and subjected to molecular docking analysis using AutoDock Vina (v1.2.0). The binding energies (kcal/mol) and interactions of key amino acids were analyzed.

### 2.12 Statistical analysis

All statistical analyses were conducted in the R programming language (v4.4.3). Differentially expressed genes were identified using Benjamini-Hochberg correction to control the false discovery rate (FDR). The performance of machine learning models was evaluated using AUC, accuracy, and OOB. For Mendelian randomization analysis, Cochran's *Q* test was used to detect heterogeneity, and the multiplicity test was performed using MR-Egger and MR-PRESSO methods. Quantitative data are presented as mean ± standard error of mean (SEM). Two-tailed Student's *t*-test was used for two group comparisons. Single-factor analysis of variance was used for comparisons among multiple data groups. Value of *p* < 0.05 was considered statistically significant.

## 3 Results

### 3.1 Identification of potential dietary intervention targets from depression-associated transcriptomes

**Figure 2:**
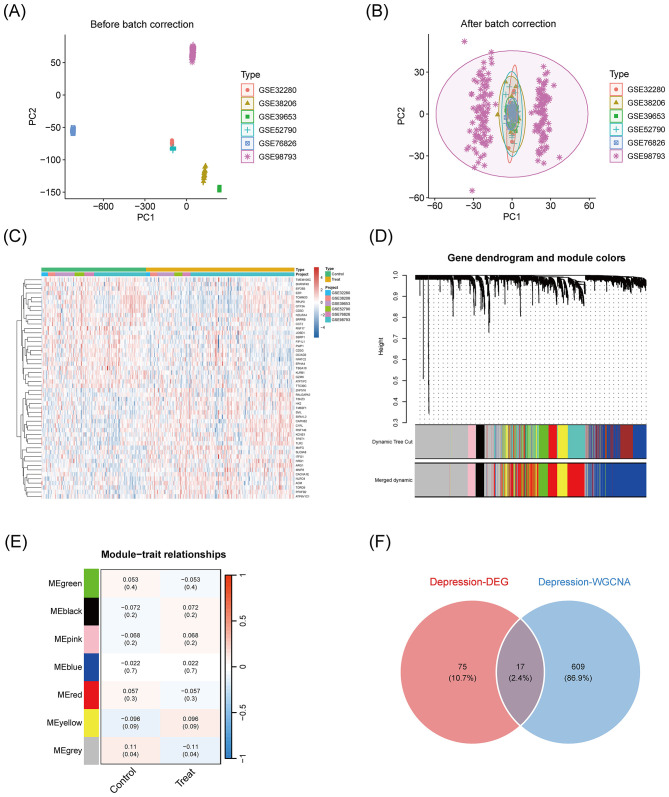
Dietary intervention target screening for depression based on transcriptome integration analysis. **(A)** Pre-batch-corrected PCA plots: dispersion distributions of the six GEO datasets (GSE32280, GSE38206, GSE39653, GSE52790, GSE76826, GSE98793) on the PC1 (−600 to 200) and PC2 (−150 to 100) axes reflecting the impact of batch effects. **(B)** Batch-corrected PCA plot: data points are clustered on the PC1 (−60 to 60) and PC2 (−40 to 40) axes, confirming the elimination of the batch effect. **(C)** Clustering heatmap of differentially expressed genes: the top 25 DEGs (ordered by *p*-value) screened based on |logFC|>0.1 and adj.*p*.Val < 0.05, demonstrating the differences in expression patterns between the disease and control groups. **(D)** WGCNA Dynamic Tree Cutting Module: gene dendrogram (height 0–1.0) with color blocks identifying co-expression modules. **(E)** Module-trait correlation analysis: only the MEGrey module was significantly and positively correlated with depression (*r* = 0.11, *p* = 0.04), the rest of the modules were not statistically significant (*p* > 0.09). **(F)** Wayne diagram of potential targets: integration of differential genes and WGCNA module genes with three components, include 75 differential genes (DEGs, 10.7%), 609 WGCNA module genes (86.9%), and 17 intersecting genes (2.4%).

To identify candidate targets for dietary intervention in depression, six GEO transcriptome datasets (GSE32280, GSE38206, GSE39653, GSE52790, GSE76826, and GSE98793) were integrated. Batch effects across datasets were assessed using principal component analysis (PCA), revealing substantial data dispersion prior to correction (PC1: −600 to 200; PC2: −150 to 100), and significant clustering following correction (PC1: −60 to 60; PC2: −40 to 40; Figures 2A, B), confirming effective removal of batch variability. Differential expression analysis using the limma package (|logFC| > 0.1; adjusted *p* < 0.05) identified 92 significant DEGs, comprising 59 upregulated and 33 downregulated genes. A heatmap of the top 25 DEGs (ranked by *p*-value) illustrated clear group-specific expression patterns (Figure 2C). Co-expression network analysis using WGCNA (dynamic cut height 0–1.0) was conducted to identify gene modules associated with depression (Figure 2D). Among the generated modules, only the Megrey module showed a statistically significant positive correlation with depression (*r* = 0.11, *p* = 0.04; Figure 2E). Integration of DEGs and WGCNA module genes via a Venn diagram (Figure 2F) identified 701 potential intervention targets, including 92 DEGs (10.7%), 609 WGCNA module genes (86.9%), and 17 overlapping genes (2.4%).

### 3.2 Functional enrichment of *Pueraria*-depression intersecting targets

**Figure 3:**
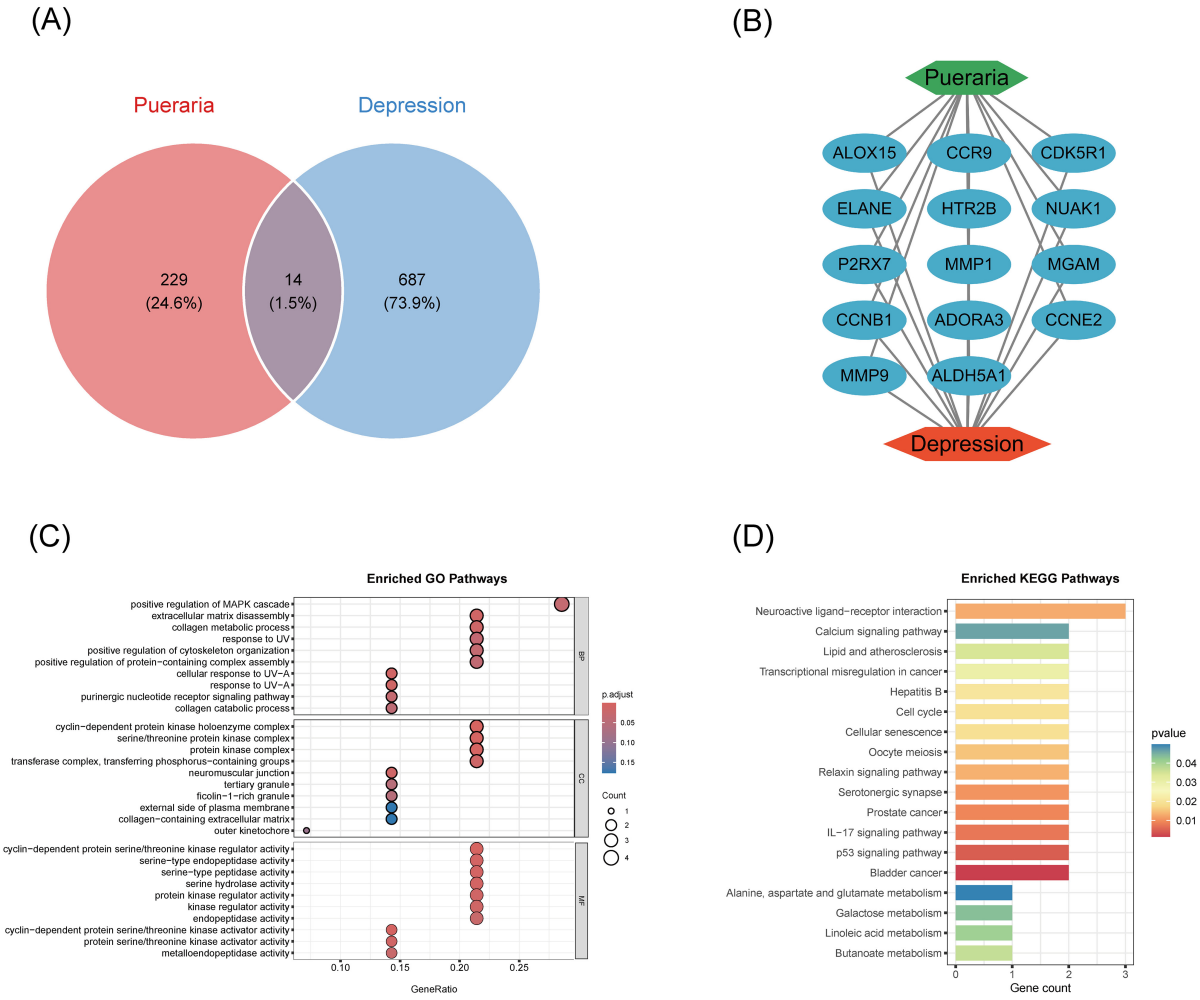
Functional enrichment analysis of *Pueraria* active constituents with intersecting targets for depression. **(A)** Wayne's diagram: showing the targets of *Pueraria* action (229, 24.6%), the targets of depressive disorders (687, 73.9%), and their intersecting genes (14, 1.5%), reflecting the overlap of the two targets. **(B)** Intersecting gene relationship network: presenting the functional interactions of 14 intersecting genes (e.g. ALDH5A1, MMP9, ADORA3, etc.). **(C)** GO enrichment analysis: dot plots demonstrating the enrichment of intersecting genes in biological processes, molecular functions, and cellular components, with GeneRatio (enriched gene ratio) on the horizontal axis and GO entries on the vertical axis, with the dot size indicating the number of enriched genes and the color depth indicating the adjusted *p*-value. **(D)** KEGG enrichment analysis: dot plot demonstrating the enrichment of intersecting genes in the signaling pathway, with Gene count (number of enriched genes) on the horizontal axis, KEGG pathway on the vertical axis, and *p*-value indicated by color.

To elucidate the mechanism by which *Pueraria* may exert antidepressant effects, 14 genes were identified at the intersection of *Pueraria*-related and depression-related targets (Figure 3A). A protein-protein interaction (PPI) network constructed from these intersecting genes revealed functional relationships (Figure 3B). GO enrichment analysis indicated significant involvement of these genes in positive regulation of the MAPK cascade, extracellular matrix catabolism, and collagen metabolic processes (Figure 3C). KEGG pathway analysis further highlighted enrichment in neuroactive ligand-receptor interactions and calcium signaling pathways (Figure 3D), underscoring potential neuromodulatory and neuroprotective mechanisms.

### 3.3 Machine learning-based identification of *Pueraria*-responsive antidepressant signature genes

**Figure 4:**
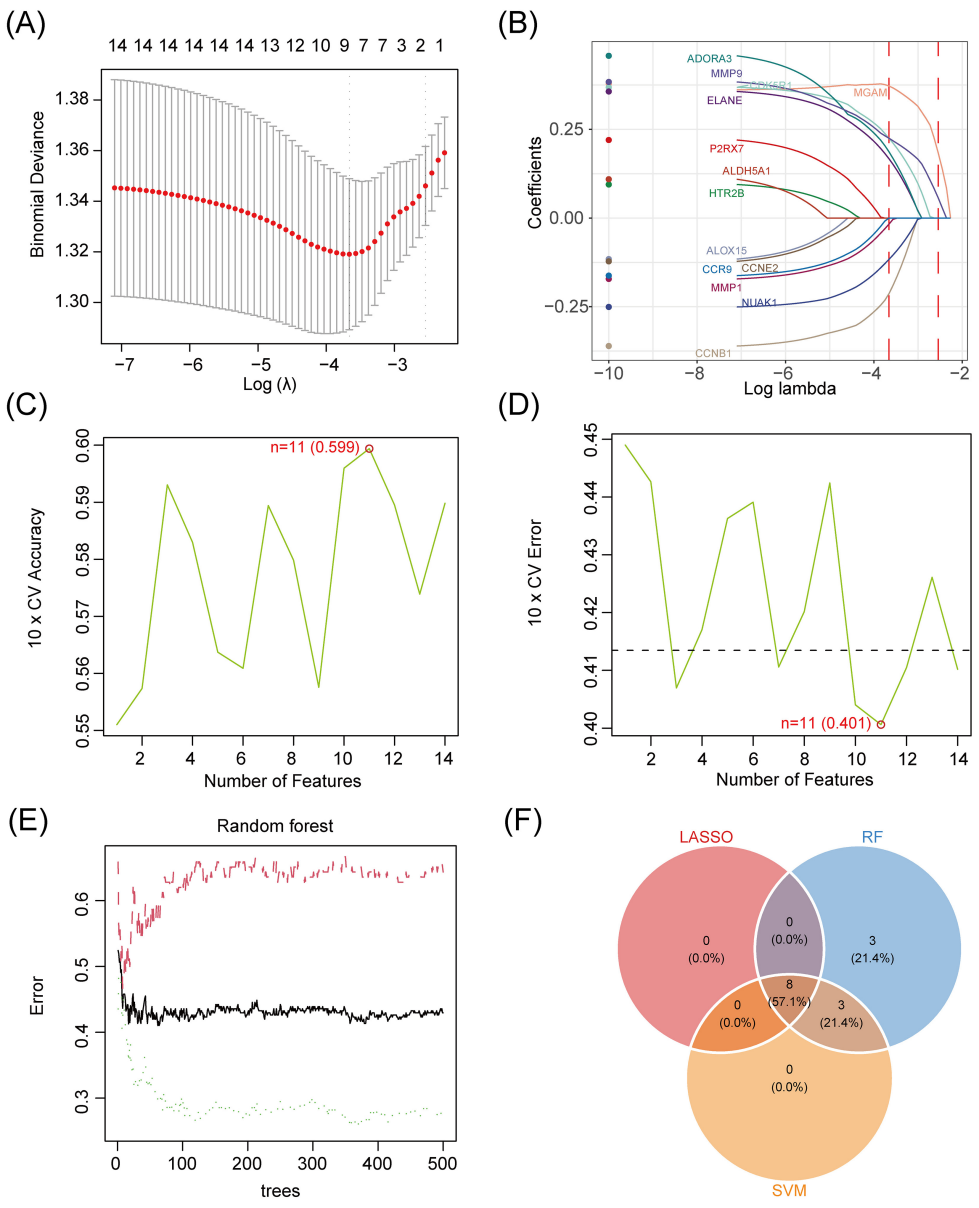
Machine learning screening of antidepressant signature genes of *Pueraria* diets. **(A)** Binomial deviation plot for the LASSO model: X-axis represents Log(λ; range −7 to −3), Y-axis represents binomial deviation (range 1.30–1.38), red dashed line marks the optimal λ-value corresponding to the minimum deviation, red dots indicate the location of the minimum deviation, and gray vertical lines show the number of features corresponding to different λ-values (1–14). **(B)** Plot of characteristic coefficients of LASSO model: X-axis represents Log λ (range −10 to −2), Y-axis represents coefficients (range −0.25 to 0.25), curves show coefficients of characteristics (e.g., ADORA3, MMP1, ELANE, PARP1, etc.) as a function of λ, and the red vertical line marks the location of the optimal λ-value. **(C)** Plot of 10-fold cross-validation accuracy of SVM model: X-axis indicates the number of features (range 2–14), Y-axis indicates the 10-fold cross-validation accuracy (range 0.55–0.60), the green dot at feature number 11 marks the highest accuracy of 0.599, and the red circle indicates the best point. **(D)** Plot of 10-fold cross-validation error for SVM model: x-axis indicates number of features (range 2–14), y-axis indicates 10-fold cross-validation error (range 0.40–0.45), green dot at number of features 11 marking the lowest error of 0.401, and red circle indicating the best point. **(E)** Error rate plot for random forest model: X-axis indicates the number of trees (range 0–500), Y-axis indicates the error rate (range 0.3–0.6), black curve shows the variation of the error rate, red curve indicates the weighted average error rate, and green dashed line indicates the variance of the error rate. **(F)** Venn diagram of feature gene selection: Circles represent the feature selection results of LASSO, RF and SVM models, with numbers labeled LASSO selects eight feature genes, RF selects 14 feature genes, SVM selects 11 feature genes, and intersection region is eight feature genes.

In order to screen the feature genes related to antidepressant of *Pueraria* diet and evaluate the performance of machine learning models, this study applied LASSO regression, support vector machine (SVM) and random forest (RF) models for feature selection. The optimal λ value was determined by 10-fold cross-validation of the LASSO model (Figure 4A) and eight feature genes were selected based on the coefficient paths (such as ADORA3 and MMP1, Figure 4B); the SVM model achieved the highest 10-fold cross-validation accuracy of 0.599 and the lowest error of 0.401 at the number of features of 11 (Figures 4C, D); and the out-of-bag error rate of the RF model with the increase in the number of trees leveled off (Figure 4E) and 14 feature genes were selected; Venn diagram integration analysis showed that the intersection of the three was 8 feature genes (Figure 4F).

### 3.4 Functional assessment via SHAP interpretation and Mendelian randomization

**Figure 5:**
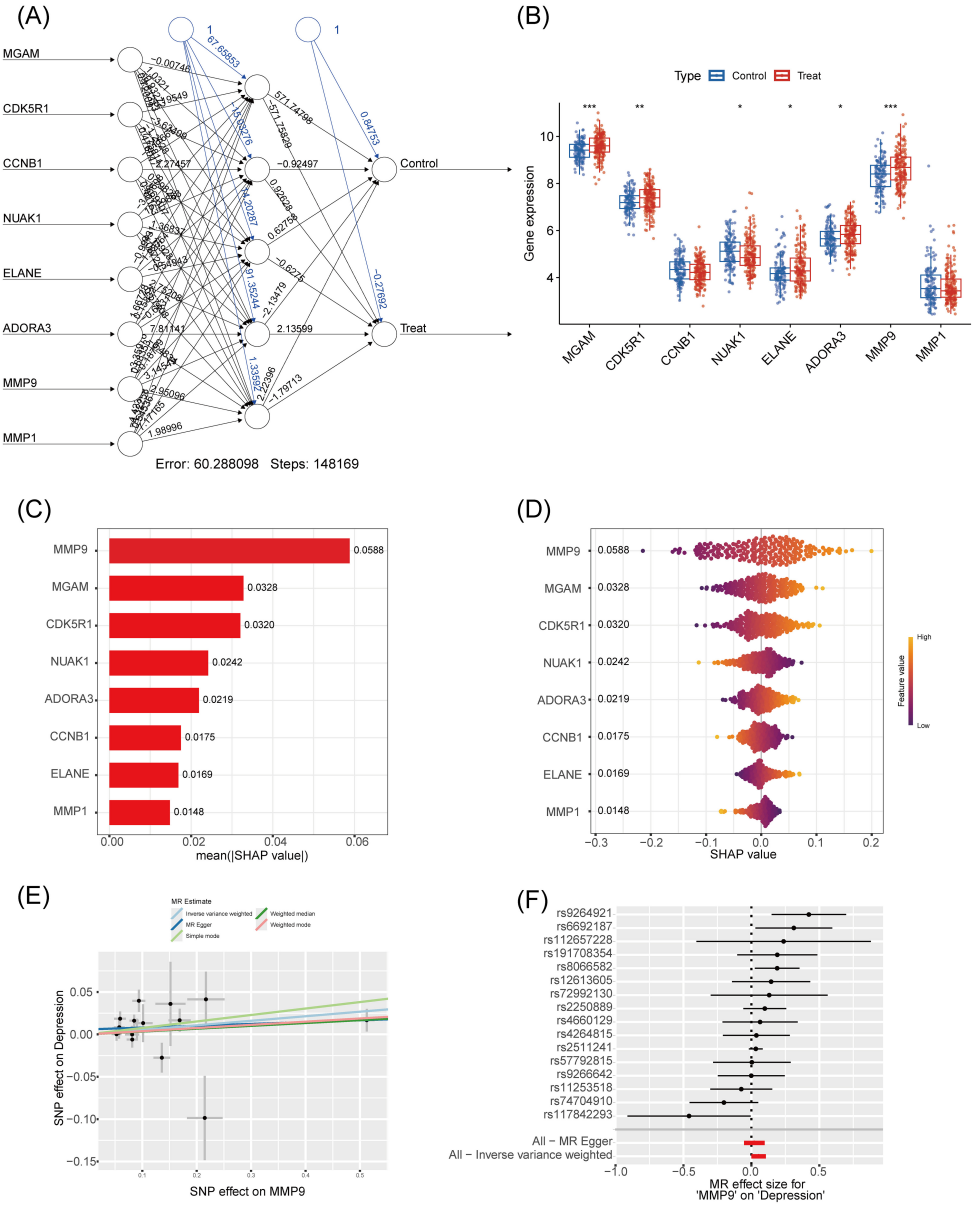
Characteristic gene depression associations based on SHAP model and Mendelian randomization. **(A)** Artificial neural network graph: input layer includes eight genes (MGAM, CDK5R1, CCNB1, NUAK1, ELANE, ADORA3, MMP9, MMP1), hidden layer with six nodes, output layer with Control and Treat, edge weights to show the strength of the association, training error of 60.288, iterations of 148,169 steps for analysis of the connection between genes and depressive states. **(B)** Gene expression box-and-line plot: Y-axis indicates gene expression values (range 4–10), X-axis for eight genes (same as **A**), box color distinguishes between Control (blue) and Treat (red), and significance markers (* indicates *p* < 0.05, ** indicates *p* < 0.01, and *** indicates *p* < 0.001) are used for comparing the expression levels. **(C)** SHAP significance feature plot): eight genes are listed on the Y-axis, mean absolute SHAP value [mean (|SHAP value|)] on the X-axis, and the bar lengths indicate the significance, which is used to quantify the magnitude of the gene's contribution to the prediction. **(D)** SHAP beeswarm plot: eight genes on the Y-axis, SHAP values on the X-axis (range −0.3 to 0.2), dot color indicates high or low gene expression (Red for high expression, blue for low expression), and the dot distribution demonstrates the direction and intensity of the effect, which is used to visualize the effect of the genes on the model output. **(E)** Mendelian randomized scatterplot: taking MMP9 as an example, the X-axis is the effect of SNP on MMP9, the Y-axis is the effect of SNP on depression, and the dots and line segments denote the effect estimates and their confidence intervals, which are used to reveal causal correlations. **(F)** Mendelian randomized effect plot: Y-axis is SNP identifier, X-axis is Mendelian randomized effect size (MR effect size), dots and line segments show effect estimates and confidence intervals of SNPs on depression for validating the causal role of MMP9.

An artificial neural network (ANN) incorporating the 8 core genes (MGAM, CDK5R1, CCNB1, NUAK1, ELANE, ADORA3, MMP9, and MMP1) was constructed to evaluate predictive value. The network achieved a training error of 60.29 after 148,169 iterations (Figure 5A), and expression analysis confirmed significant differences in these genes between depression and control groups (Figure 5B). SHAP analysis identified MMP9 as the most important feature (SHAP value = 0.0588), followed by MGAM (0.0328; Figure 5C). Swarm plots further illustrated the nuanced effects of gene expression on model predictions (Figure 5D). Mendelian randomization (MR) analysis using genome-wide SNP data confirmed a potential causal link between MMP9 expression and depression (Figures 5E, F), reinforcing its role as a therapeutic target.

### 3.5 Molecular docking validates binding affinities of *Pueraria* compounds to target genes

**Figure 6:**
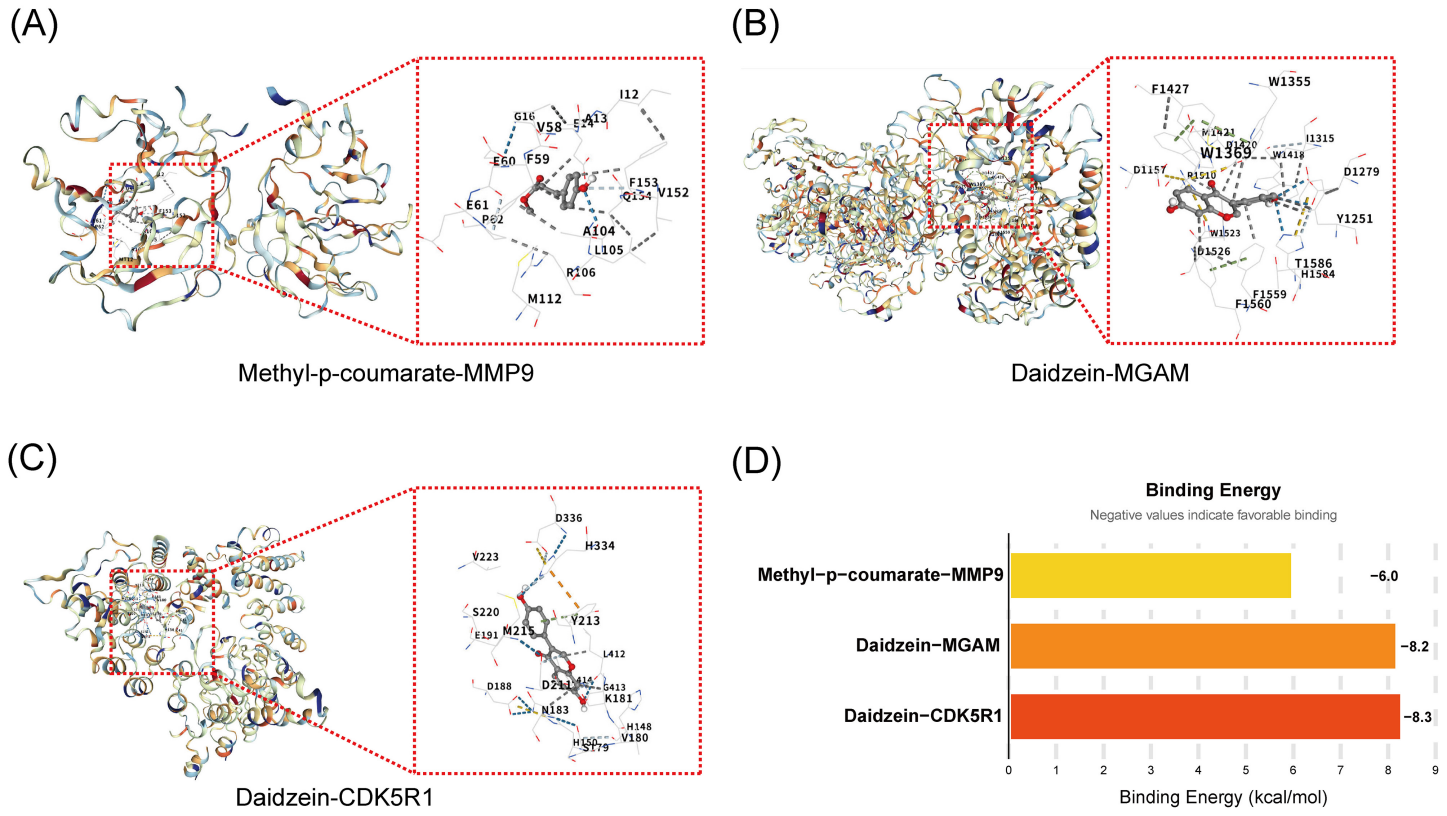
Validation of molecular docking binding affinity of active components of *Pueraria* with characterized genes. **(A)** The binding diagram of methyl p-coumarate and MMP9: Showing the molecular docking structure with key amino acids including G61, V58, E59, F153, etc., where G61 and V58 may form a hydrogen bond, which is used to visualize the binding site and interaction pattern. **(B)** The binding diagram of daidzein with MGAM: Demonstrating the docked conformation, key amino acids including D145, F1427, W1369, etc., W1369, etc. are stabilized by hydrophobic interactions and are used to analyze the binding stability. **(C)** The binding diagram of daidzein with CDK5R1:Presenting the docking interface, key amino acids including V223, D336, H334, etc. H334 may be involved in hydrogen bonding for resolving the binding region and type of force. **(D)** Binding Energy Analysis Plot: The X-axis is labeled with Compound-Gene Pairs (Methyl p-coumarate-MMP9, Daidzein-MGAM, Daidzein-CDK5R1), and the Y-axis represents Binding Energy (unit: kcal/mol). The bar height corresponds to energy values, where negative values indicate favorable binding. This plot quantifies affinity strength and enables comparison.

In order to verify the binding affinities of the nutrient active components in *Pueraria* with the antidepressant signature genes to elucidate their molecular mechanisms of action, the present study used molecular docking technology to analyze the interactions of the key components (DD, MpC) with the signature genes (MMP9, MGAM, CDK5R1). The binding sites were revealed by docking simulations (Figures 6A–C) and the binding energies were quantified (Figure 6D); The experimental results showed that the binding of methyl p-coumarate to MMP9 involves key amino acids with a binding energy of −6.0 kcal/mol; the binding of DD to MGAM relies on hydrophobic interactions with an energy of −8.2 kcal/mol; the binding of DD to CDK5R1 involves hydrogen bonds with an energy of −8.3 kcal/mol (negative values indicate stable binding, with DD-CDK5R1 exhibiting the strongest affinity).

### 3.6 *Pueraria*-derived compounds reverse depression-like behavior in CMS mice

**Figure 7:**
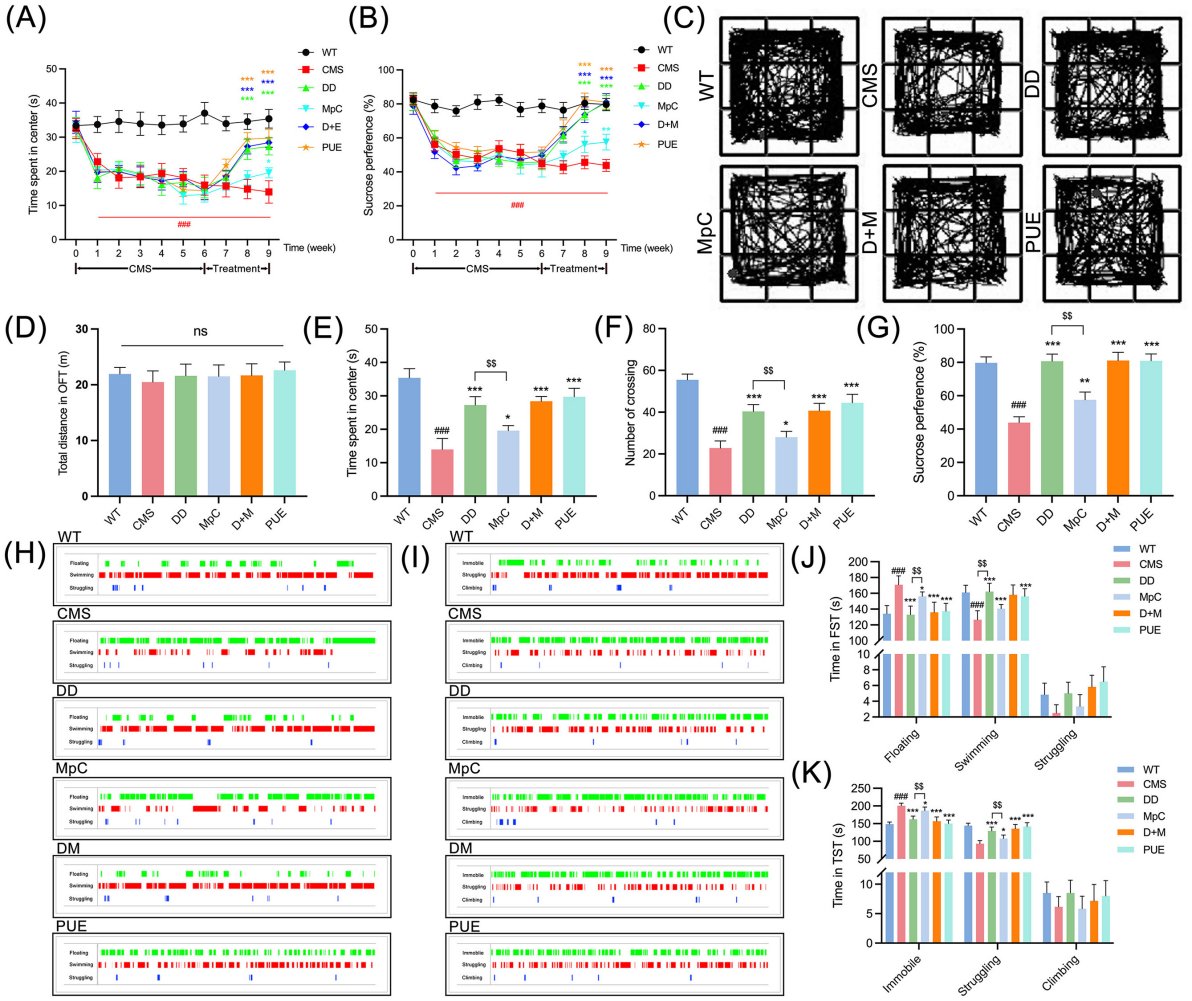
Active components of *Pueraria* ameliorate depression-like behavior in CMS mice. **(A, B)** In the OFT and SPT, CMS mice spent significantly less time in the center region **(A)** and preferred sucrose **(B)** than wild mice, while DD, McP, D+M, PUE have opposite effects on anxiety behavior in mice relative to CMS mice (*n* = 6). **(C–F)** Representative action trajectories of mice **(C)** were plotted at week 9 to record the total distance of the OFT **(D)**, the time spent in center **(E)**, and the number of crossing **(F)** was traversed. **(G)** Quantitative assessment of the sucrose preference in SPT in mice at week 9. **(H, I)** Representative states of FST **(H)**, TST **(I)** of mice were plotted at week 9. **(J)** Quantitative assessment of the floating, swimming and struggling time in mice in FST. **(K)** Quantitative assessment of the immobile, struggling, and climbing time in mice in the TST. All values were presented as mean ± SEM; ^$^*p* < 0.01. ^###^*p* < 0.001 compared with WT group. *p < 0.05, **p < 0.01, ***p < 0.001 compared with CMS group.

To confirm the antidepressant effects of *Pueraria*-derived compounds, CMS was induced in mice for 6 weeks, leading to stable depression-like phenotypes. Subsequently, mice were treated with vehicle, DD, MpC, DD+MpC, or PUE for 3 weeks. Behavioral assessments via the OFT and SPT demonstrated significant reversal of depression-like behaviors in the DD, MpC, and PUE treatment groups (Figures 7A, B). OFT showed increased exploratory behavior in treated mice (Figure 7C), supported by elevated center time and crossing frequency (Figures 7E, F), without signs of locomotor impairment (Figure 7D). According to SPT, treatment with the active component of *Pueraria* significantly boosted the mice's preference for sucrose (Figure 7G). Moreover, FST and TST results confirmed significantly reduced immobility and increased swimming/struggling times in treated groups compared to CMS controls (Figures 7H–K). Notably, DD treatment exhibited the most pronounced therapeutic effect, suggesting it may be a leading candidate for dietary-based depression intervention.

## 4 Discussion

Traditional pharmacology has long served as the cornerstone of drug discovery and mechanistic research, providing a well-established framework for elucidating the effects of single compounds on specific molecular targets or pathological phenotypes. However, this reductionist approach—which relies heavily on *in vivo* and *ex vivo* models—is associated with several limitations, including high cost, time inefficiency, and high failure rates, particularly in the context of complex, multifactorial disorders such as cancer, Alzheimer's disease, and depression (22). Moreover, it often fails to capture the synergistic effects inherent to multi-component therapeutics, such as those employed in traditional Chinese medicine (TCM) (23). In contrast, network pharmacology—emerging from the integration of systems biology and modern pharmacology—provides a holistic framework for drug discovery. This approach conceptualizes diseases as complex networks of interacting genes, proteins, and pathways, thereby facilitating the identification of multi-target drug actions through the systematic integration of compound-target-disease relationships (24). In the present study, we employed this methodology to investigate the antidepressant potential of *Pueraria*, a component of TCM, using a multi-step computational and analytical pipeline.

We applied a network pharmacology approach to screen 701 potential targets from a GEO transcriptome dataset related to dietary interventions for depression, ultimately identifying 14 overlapping genes between *Pueraria* and depression. GO and KEGG enrichment analyses revealed that these targets were significantly associated with positive regulation of the MAPK cascade, extracellular matrix catabolism, collagen metabolic processes, neuroactive ligand-receptor interactions, and calcium signaling pathways. Existing evidence indicates that the MAPK signaling pathway (25), remodeling of the neuronal extracellular matrix (26), neuroactive ligand-receptor interactions (27), and dysregulation of neuronal calcium signaling (28) are closely implicated in the pathogenesis of depression. To further refine the key targets, we applied LASSO regression, SVM and RF models, which identified eight characteristic genes: MGAM, CDK5R1, CCNB1, NUAK1, ELANE, ADORA3, MMP9, and MMP1. Among these, MMP9, MGAM, and CDK5R1 emerged as the most prominent and promising therapeutic targets, supporting the hypothesis that the active components of *Pueraria* exert antidepressant effects through multi-pathway and multi-target mechanisms.

MMP9 is physiologically expressed in neurons, astrocytes, and microglia within the central nervous system. Its aberrant elevation has been strongly linked to depression (29), and minocycline—a tetracycline antibiotic and MMP-9 inhibitor—has been proposed as a therapeutic agent for depressive symptoms (30, 31). CDK5R1, also known as p35, is a specific activator of CDK5. Dysregulation of CDK5 directly impairs neurotransmission and synaptic plasticity (32), and the CDK5/p35 complex has been shown to play a key role in modulating depressive-like behaviors and antidepressant responses (33). MGAM, meanwhile, is involved in biological processes related to oxidative stress and inflammation and has been proposed as a potential diagnostic and prognostic biomarker for patients with pain-depression comorbidity (34). Molecular docking analysis confirmed interactions between these three key targets and the active components of *Pueraria*, revealing binding sites with DD and MpC. DD exhibited the strongest binding affinity with CDK5R1. Previous studies have reported that puerarin (A type of isoflavone) reduces the incidence and progression of depression and demonstrates neuroprotective effects in various neurological disorders (16, 35, 36). DD is also a type of isoflavone, primarily found in leguminous plants and is known to regulate hormone secretion, protein synthesis, and growth factor activity (37). It has been shown to exert protective effects on hippocampal neurons, ameliorate cognitive deficits, improve memory capacity, promote hippocampal neurogenesis, and display a range of pharmacological activities within the central nervous system (38, 39).

Although methyl p-coumarate—a type of aromatic acid derivative present in *Pueraria* at low concentrations—has not been previously reported to possess neuroprotective properties, it exhibits significant anti-inflammatory activity (40, 41). Its potential role in mitigating neuroinflammation warrants further investigation. Behavioral tests, including OFT, SPT, FST, and TST, conducted in the present study indicated that both DD and PUE almost completely reversed depressive-like behaviors in a CMS mouse model (with no statistically significant differences between the two treatments), whereas methyl p-coumarate showed a considerably weaker antidepressant effect. DD mitigates oxidative stress by inhibiting NOX-4, reducing ROS production, and preserving antioxidant enzymes. This mechanism mitigates neural damage and enhances nerve conduction velocity (42). Additionally, DD activates the proliferator-activated receptor-γ, modulating synaptic function to achieve neuroprotective effects (43). Arginase 1 protects motor neurons from nutrient deprivation and enables sensory neurons to overcome the inhibitory effects of myelin proteins on neurite outgrowth. DD has been clinically established as a safe Arginase 1 transcriptional inducer that can directly cross the blood-brain barrier and exert its effects effectively without requiring pretreatment (44). The above factors may account for the differences in antidepressant efficacy between DD and MpC. Based on our network pharmacology analysis, we propose that these neuroprotective and antidepressant effects may arise through CDK5R1-mediated downregulation of CDK5 activity in the dentate gyrus (DG) subregion of the hippocampus (33), and/or through MGAM involvement in the regulation of oxidative stress and neuroinflammation (34).

This study employed network pharmacology methods to screen potential antidepressant components in *Pueraria*. Initial screening was based on oral bioavailability (OB) and drug-like properties (DL) parameters from the TCMSP database. The established screening criteria (OB ≥ 30% and DL ≥ 0.18) are widely adopted and effectively focus on components with favorable pharmacokinetic properties, thereby enhancing the efficiency of discovering drug-like lead compounds (45). However, these criteria have limitations. Certain natural products with low oral bioavailability or unique structures may still exert therapeutic effects through indirect mechanisms, including intestinal metabolism, action on peripheral targets, or gut microbiota regulation (46). Consequently, current standards may not capture all bioactive compounds in *Pueraria*. Despite these limitations, the key components identified using these criteria demonstrated significant antidepressant activity in *in vivo* experiments, validating the effectiveness of this screening strategy for identifying core active ingredients. Future studies should consider employing more lenient screening criteria or integrating complementary experimental techniques, such as metabolomics, to more comprehensively elucidate the antidepressant mechanisms of *Pueraria*.

Although network pharmacology provides a powerful, holistic framework for predicting drug synergies and mechanisms at a systems level, which aligns well with the complex pathology of depression, further experimental validation is essential to fully realize the therapeutic potential of *Pueraria's* multi-target, multi-pathway approach. Subsequent *in vivo* and *ex vivo* studies should focus on validating the eight characteristic gene targets, including MMP9, MGAM, and CDK5R1. It is important to note that public transcriptome datasets may not fully capture the heterogeneity of depression across patient populations, and CMS models are limited by species differences and incomplete symptom recapitulation. Therefore, rigorously designed, multi-faceted *in vivo* and *ex vivo* studies will be critical for further investigating the pharmacokinetics, bioavailability, and mechanistic underpinnings of *Pueraria* and its active constituents.

## 5 Conclusion

In this study, we employed an integrated approach combining network pharmacology and machine learning to identify key targets—CDK5R1, MGAM, and MMP9—underlying the antidepressant effects of *Pueraria's* active ingredients. Molecular docking analyses revealed that the primary bioactive compounds in *Pueraria*, daidzein and methyl p-coumarate, exhibit strong binding affinity with these characterized targets. The antidepressant efficacy of these compounds was further corroborated through *in vivo* experiments using a CMS mouse model. This methodology provides a powerful framework for the systematic exploration of *Pueraria's* mechanisms of action in the treatment of depression. However, it should be noted that the current findings are based on computational predictions and initial *in vivo* validation; further experimental studies are required to fully elucidate the pharmacological pathways involved. In summary, our results suggest that *Pueraria* represents a promising and sustainable complementary intervention for depression. Its incorporation into dietary regimens may offer a viable strategy for ameliorating depression-like behaviors.

## Data Availability

The original contributions presented in the study are included in the article/supplementary material, further inquiries can be directed to the corresponding authors.
